# Umbilical Nodule in a Patient With a History of Colon Cancer

**DOI:** 10.7759/cureus.106029

**Published:** 2026-03-28

**Authors:** Luis G García-Guzmán, Gerardo S Caussade-Silvestrini, Cristina P Gerena-Maldonado, Fabiola Pabón-González, Melanie D Medina-Figueroa, Xavier Sánchez-Flores

**Affiliations:** 1 Department of Dermatology, University of Puerto Rico, Medical Sciences Campus, San Juan, USA

**Keywords:** colorectal cancer, cutaneous metastasis, immunohistochemistry, sister mary joseph nodule, umbilical metastasis

## Abstract

A Sister Mary Joseph nodule (SMJN) is a rare cutaneous manifestation of metastatic intra-abdominal or pelvic malignancy and typically indicates advanced disease. We report the case of a 48-year-old female patient who presented to our dermatology clinic with a three-month history of a painless, skin-colored umbilical mass with intermittent foul-smelling serosanguinous drainage. Her medical history was noteworthy for metastatic adenocarcinoma of the colon diagnosed five years earlier, initially treated with subtotal colectomy and adjuvant chemotherapy, followed by recurrence with ovarian involvement consistent with a Krukenberg tumor and subsequent immunotherapy-induced remission. Physical examination and histopathologic findings were consistent with the diagnosis of an SMJN of colorectal origin, supported by immunohistochemical staining confirming metastatic adenocarcinoma of the colon. This case highlights the diagnostic value of a dermatologic evaluation and a timely biopsy in identifying SMJN and guiding appropriate staging and management.

## Introduction

The Sister Mary Joseph nodule (SMJN) is an atypical yet significant clinical finding, described as an umbilical metastasis that typically arises from intra-abdominal or pelvic malignancies [1]. These nodules are reported to be present in approximately 1%-3% of patients with an abdominopelvic malignancy [2]. The term “Sister Mary Joseph nodule” was popularized by Dr. Hamilton Bailey, an English surgeon, in 1949 to honor Sister Mary Joseph, who first observed the association between umbilical nodules and visceral cancers during abdominal surgeries [3]. The clinical presentation of SMJNs varies but often includes a palpable umbilical mass with overlying skin changes, including crusting, ulceration, or discharge [3]. Dermoscopic findings, such as polymorphic vessels and milky-red regions in visible umbilical masses, may justify diagnostic biopsies [4]. Accordingly, some cases are identified during routine dermatologic exams, emphasizing their importance. Despite their rarity, SMJNs represent an important clinical finding, as they are commonly associated with advanced metastatic disease and poor prognosis, highlighting the critical role of dermatologic recognition in prompt diagnosis and management [1,5]. In this context, we present the case of an umbilical nodule in a patient with a history of metastatic colon cancer, evaluated at the dermatology clinics and ultimately diagnosed with an SMJN following a biopsy.

## Case presentation

A 48-year-old woman presented to our dermatology clinics with a three-month history of a painless, skin-colored umbilical mass that intermittently drained a foul-smelling fluid and had progressively increased in size over time. The lesion was initially subtle and asymptomatic but later became more noticeable due to persistent malodorous serosanguinous discharge and surface changes, prompting a dermatologic evaluation.

Her medical history was noteworthy for chronic, unremitting abdominal pain and an extensive oncologic course associated with metastatic adenocarcinoma of the colon, diagnosed five years prior to her visit to our clinic and treated with subtotal colectomy. She achieved an initial remission after receiving adjuvant systemic chemotherapy; however, the malignancy recurred approximately one year later. At that time, exploratory surgery revealed ovarian involvement consistent with a Krukenberg tumor, which was surgically removed. She subsequently received adjuvant immunotherapy, although specific details of the regimen were not available, resulting in a second period of clinical remission lasting approximately 3.5 years, after which the current umbilical lesion developed.

**Figure 1:**
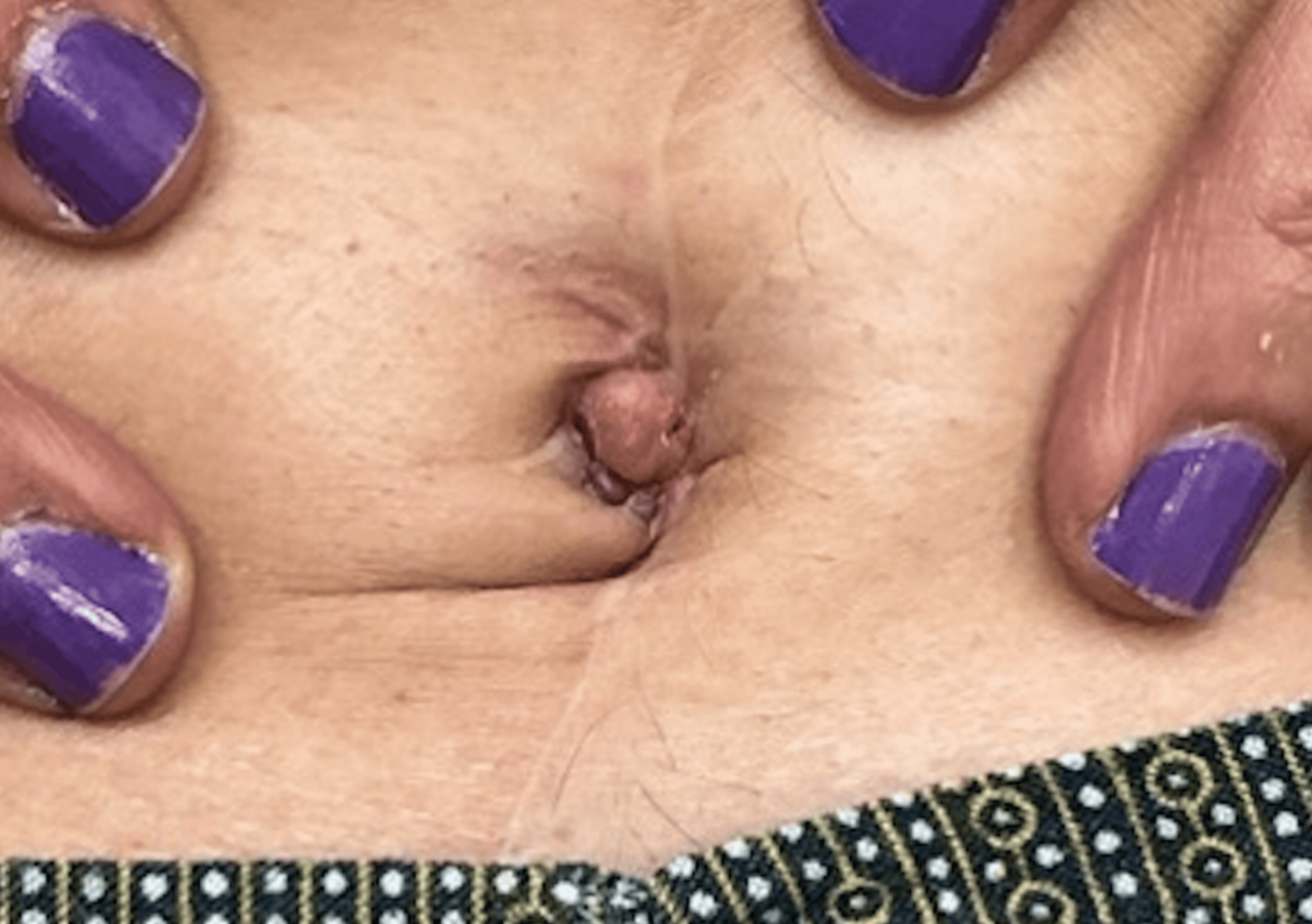
A 1.5 cm skin-colored nodule with crust on the umbilical area in a patient with prior colon carcinoma.

**Figure 2:**
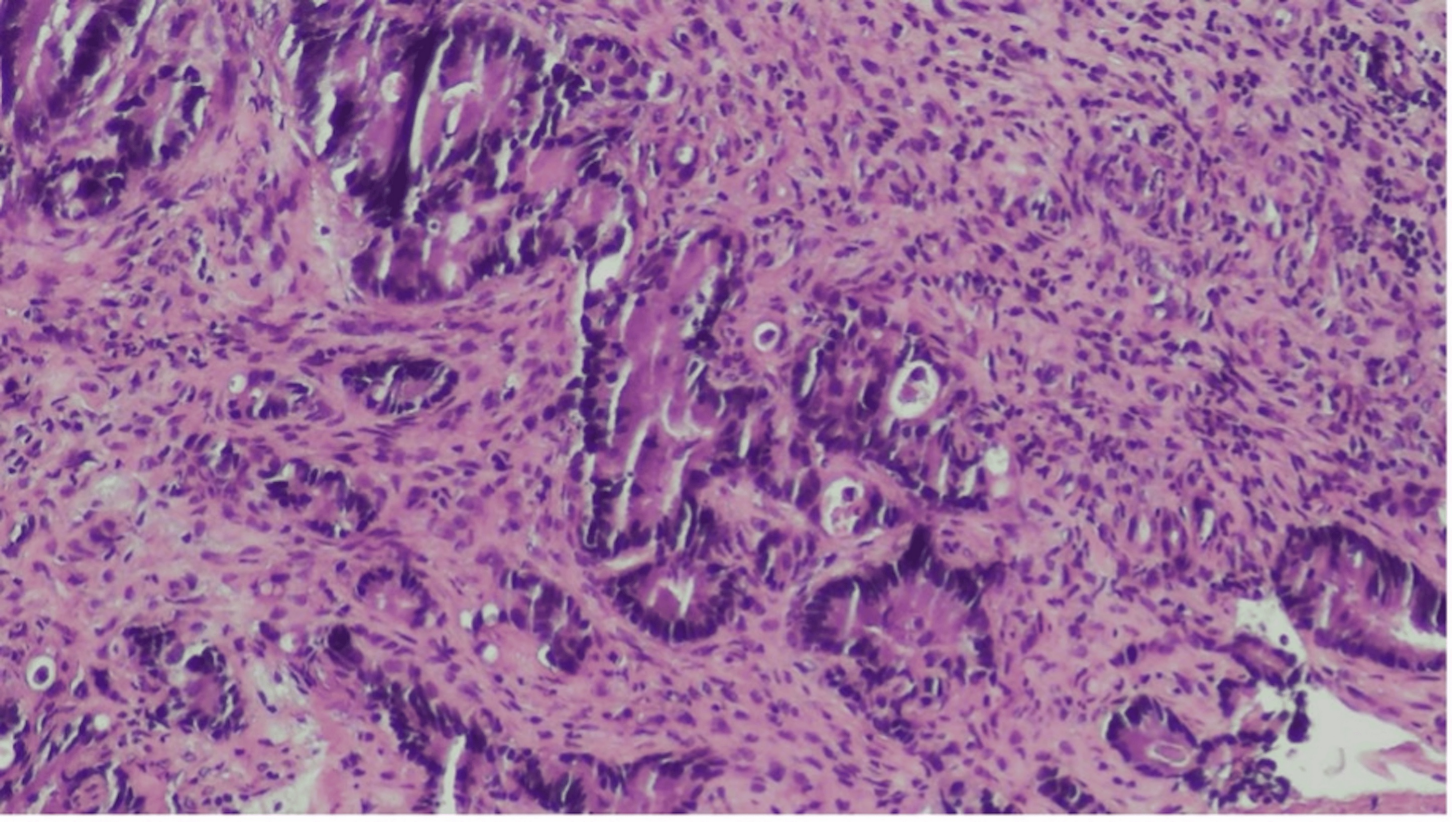
Histopathologic image (hematoxylin-eosin, ×100) showing atypical ductal structures infiltrating dermal collagen bundles, consistent with metastatic adenocarcinoma.

Upon examination in our dermatology clinic, a 1.5 cm skin-colored nodule with a crust in the umbilical area was noted (Figure 1). At presentation, differential diagnoses included an SMJN, umbilical endometriosis (Villar’s nodule), primary umbilical adenocarcinoma, and benign lesions such as epidermal inclusion cysts and granulomas. Given her extensive oncologic history, a punch biopsy of the lesion was performed to evaluate for a possible cutaneous metastasis. Histopathologic examination of the umbilical biopsy revealed proliferation of atypical ductal structures between collagen bundles of the dermis (Figure 2).

**Figure 3:**
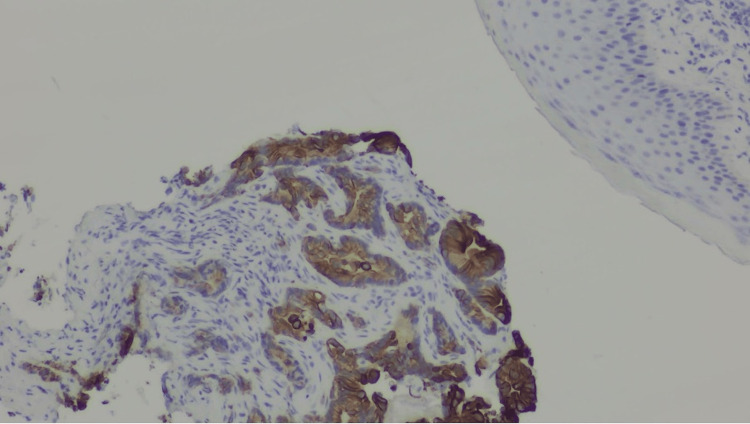
Immunohistochemical staining demonstrating strong CK20 positivity in tumor cells, supporting a colorectal origin of the metastatic lesion.

Immunohistochemistry demonstrated CK20-positive tumor cells (Figure 3), consistent with colon cancer. Immunohistochemical tests showed tumor cells positive for AE1/AE3, EMA, and CEA, and negative for CK7 and CK5/6, confirming metastatic colon adenocarcinoma. Based on the clinical presentation, biopsy results, and immunochemistry, a diagnosis of SMJN was established. In our case, the patient had a known colon cancer that was ultimately identified as the source of the metastatic SMJN.

A subsequent positron emission tomography (PET) scan revealed metastatic lesions in the liver, lungs, umbilicus, and inguinal lymph nodes. The patient was restarted on systemic therapy, including chemotherapy and radiotherapy, and was initiated on regorafenib. However, follow-up data regarding treatment response were not available at the time of this report.

## Discussion

An SMJN, such as the one presented by our patient, is an uncommon finding that may be the initial sign of an underlying malignancy, underscoring the importance of heightened clinical suspicion [1,5]. Diagnostic techniques, such as computed tomography (CT) scans and skin biopsies, are necessary not only to confirm the diagnosis but also to evaluate the extent of metastatic disease [6]. The primary tumors most frequently involved include gastric, colorectal, pancreatic, and ovarian cancers, with ovarian cancer being most common in women and colorectal cancer in men [5,7]. Therefore, when examined, most nodules are adenocarcinomas, primarily originating from gastrointestinal and gynecological tumors [5,7].

The prognosis for patients with SMJN is generally poor, with median survival ranging from two to 11 months without treatment, with some reports stating that median survival is less than eight months [5,6]. However, other studies account that patients may live up to 21 months or longer from the time of diagnosis, depending on the tumor’s origin, stage, and treatment [5,7,8]. Surgery, combined with chemotherapy, has been used in selected cases, while radiotherapy is usually intended for patients with widespread metastases [6,9]. Newer targeted agents, such as regorafenib for metastatic colorectal cancer, may offer modest survival benefit [10]. Accordingly, this therapeutic agent was added to our patient’s regimen by her oncologist. Nonetheless, in cases of extensive disease, treatment is mainly palliative, focusing on symptom control and systemic chemotherapy [9,11]. Although rare, SMJN remains a warning sign of advanced malignancy with essential implications for staging, prognosis, and treatment strategies.

In addition to guiding prognosis and treatment, an accurate diagnosis of SMJN requires distinguishing it from other umbilical lesions that may share overlapping clinical and histopathologic features. Differential diagnoses of SMJN include umbilical endometriosis (also known as Villar’s nodule), primary umbilical adenocarcinoma, and benign lesions. Benign lesions, such as epidermal inclusion cysts or granulomas, were ruled out from the outset in our case because malignant cells were identified on the umbilical biopsy. Umbilical endometriosis, or Villar’s nodule, is characterized by endometrial glands surrounded by stroma on histopathological examination [12]. Hence, this diagnosis was less likely in our case. On the other hand, a primary umbilical adenocarcinoma may be positive for CK7 and CK19 and negative for CK20 [13]. Although variations in cytokines may exist and, on their own, may not rule out this entity in our patient, negative colonoscopy, gastroscopy, magnetic resonance cholangiopancreatography, PET scan, and thoracic and abdominopelvic CT scans are needed for this type of cancer to be considered [13]. Since our patient had a PET scan and a colonoscopy that were remarkable for cancer, this disease was ruled out.

Ultimately, our patient was confirmed to have an SMJN due to her entire clinical background and immunohistochemical profile, which included a marker pattern of CK20⁺/CK7⁻ associated with her primary tumor of colorectal origin [14]. Due to the complexity and length of her underlying disease, she remains under multidisciplinary care and evaluation.

## Conclusions

This interesting case highlights the enduring diagnostic importance of cutaneous manifestations as visible indicators of underlying internal malignancy, particularly in patients with a known history of advanced or previously metastatic cancer. The development of an umbilical lesion in this clinical context should prompt immediate concern for an SMJN, as its presence often reflects widespread disease, carries prognostic implications, and must be known before deciding on the patient’s therapy. Hence, prompt recognition of this entity is crucial, as it should trigger a comprehensive evaluation for both the primary tumor and additional sites of metastatic spread. Early dermatology consultation, timely skin biopsy, and appropriate immunohistochemical studies are essential to establish an accurate diagnosis, guide oncologic decision-making, and facilitate coordinated multidisciplinary management to optimize patient care and outcomes.

## References

[1] Segovis CM, Dyer RB (2017). The "Sister Mary Joseph nodule". Abdom Radiol (NY).

[2] Leyrat B, Bernadach M, Ginzac A, Lusho S, Durando X (2021). Sister Mary Joseph nodules: a case report about a rare location of skin metastasis. Case Rep Oncol.

[3] Sina B, Deng A (2007). Umbilical metastasis from prostate carcinoma (Sister Mary Joseph's nodule): a case report and review of literature. J Cutan Pathol.

[4] Gracia-Darder I, Del Pozo Hernando LJ (2022). Sister Mary Joseph's nodule. J Cutan Med Surg.

[5] Hugen N, Kanne H, Simmer F (2021). Umbilical metastases: real-world data shows abysmal outcome. Int J Cancer.

[6] Palaniappan M, Jose WM, Mehta A, Kumar K, Pavithran K (2010). Umbilical metastasis: a case series of four Sister Joseph nodules from four different visceral malignancies. Curr Oncol.

[7] Majdoubi A, Bouhout T, Harhar M, Mirry A, Badr S, Harroudi TE (2021). Radical treatment of Sister Mary-Joseph nodule: case report and literature review. Pan Afr Med J.

[8] Gabriele R, Conte M, Egidi F, Borghese M (2005). Umbilical metastases: current viewpoint. World J Surg Oncol.

[9] Sasai K, Kawamura M, Okumura K, Kawai Y (2024). Radiation therapy for Sister Mary Joseph's nodule: a review. Adv Radiat Oncol.

[10] Novakova-Jiresova A, Kopeckova K, Boublikova L (2020). Regorafenib for metastatic colorectal cancer: an analysis of a registry-based cohort of 555 patients. Cancer Manag Res.

[11] Larentzakis A, Theodorou D, Fili K, Manataki A, Bizimi V, Tibishrani M, Katsaragakis S (2008). Sister Mary Joseph's nodule: three case reports. Cases J.

[12] Panicker R, Pillai N, Nagarsekar U (2010). Villar's nodule: a rare presentation of external endometriosis. Med J Armed Forces India.

[13] Febrero B, Ruiz de Angulo D, Ortiz MÁ, López MJ, Parrilla P (2014). Primary adenocarcinoma of the navel: an uncommon entity. Cir Esp.

[14] Al-Maghrabi J, Emam E, Gomaa W (2018). Immunohistochemical staining of cytokeratin 20 and cytokeratin 7 in colorectal carcinomas: four different immunostaining profiles. Saudi J Gastroenterol.

